# Adenylate Kinase 4—A Key Regulator of Proliferation and Metabolic Shift in Human Pulmonary Arterial Smooth Muscle Cells via Akt and HIF-1α Signaling Pathways

**DOI:** 10.3390/ijms221910371

**Published:** 2021-09-26

**Authors:** Magdalena Wujak, Christine Veith, Cheng-Yu Wu, Tessa Wilke, Zeki Ilker Kanbagli, Tatyana Novoyatleva, Andreas Guenther, Werner Seeger, Friedrich Grimminger, Natascha Sommer, Ralph Theo Schermuly, Norbert Weissmann

**Affiliations:** 1 Universities of Giessen and Marburg Lung Center (UGMLC), German Center for Lung Research (DZL), Excellence Cluster Cardio-Pulmonary Institute (CPI), Justus-Liebig University, 35392 Giessen, Germany; Christine.Veith@innere.med.uni-giessen.de (C.V.); Cheng-Yu.Wu@innere.med.uni-giessen.de (C.-Y.W.); Tessa.Wilke@innere.med.uni-giessen.de (T.W.); Zeki.I.Kanbagli@innere.med.uni-giessen.de (Z.I.K.); Tatyana.Novoyatleva@innere.med.uni-giessen.de (T.N.); Andreas.Guenther@innere.med.uni-giessen.de (A.G.); Werner.Seeger@innere.med.uni-giessen.de (W.S.); Friedrich.Grimminger@innere.med.uni-giessen.de (F.G.); Natascha.Sommer@innere.med.uni-giessen.de (N.S.); Ralph.Schermuly@innere.med.uni-giessen.de (R.T.S.); 2 Department of Medicinal Chemistry, Collegium Medicum in Bydgoszcz, Faculty of Pharmacy, Nicolaus Copernicus University in Toruń, 85-089 Bydgoszcz, Poland; 3 Department of Internal Medicine, Universities of Giessen and Marburg Lung Center (UGMLC), Institute for Lung Health (ILH), Cardio-Pulmonary Institute (CPI), German Center for Lung Research (DZL), 35392 Giessen, Germany; 4 Department of Lung Development and Remodelling, Max-Planck Institute for Heart and Lung Research, 61231 Bad Nauheim, Germany

**Keywords:** adenylate kinase, AK4, PASMCs, hypoxia, HIF-1α, pulmonary hypertension, metabolic shift

## Abstract

Increased proliferation of pulmonary arterial smooth muscle cells (PASMCs) in response to chronic hypoxia contributes to pulmonary vascular remodeling in pulmonary hypertension (PH). PH shares numerous similarities with cancer, including a metabolic shift towards glycolysis. In lung cancer, adenylate kinase 4 (AK4) promotes metabolic reprogramming and metastasis. Against this background, we show that AK4 regulates cell proliferation and energy metabolism of primary human PASMCs. We demonstrate that chronic hypoxia upregulates AK4 in PASMCs in a hypoxia-inducible factor-1α (HIF-1α)-dependent manner. RNA interference of AK4 decreases the viability and proliferation of PASMCs under both normoxia and chronic hypoxia. AK4 silencing in PASMCs augments mitochondrial respiration and reduces glycolytic metabolism. The observed effects are associated with reduced levels of phosphorylated protein kinase B (Akt) as well as HIF-1α, indicating the existence of an AK4-HIF-1α feedforward loop in hypoxic PASMCs. Finally, we show that AK4 levels are elevated in pulmonary vessels from patients with idiopathic pulmonary arterial hypertension (IPAH), and AK4 silencing decreases glycolytic metabolism of IPAH-PASMCs. We conclude that AK4 is a new metabolic regulator in PASMCs interacting with HIF-1α and Akt signaling pathways to drive the pro-proliferative and glycolytic phenotype of PH.

## 1. Introduction

Hypoxia is a condition in which cells or tissues are deprived of adequate oxygen supply. Hypoxia-inducible factors (HIFs) are the oxygen-sensing transcription factors, orchestrating physiological responses important for tissue protection and adaptation under hypoxic stress [1,2]. The activation of HIF-1α is the central event of hypoxic signaling, resulting in downstream gene regulation and promoting a metabolic shift (switch) in energy metabolism from mitochondrial oxidative phosphorylation (OXPHOS) towards glycolysis, thus adapting cells to anaerobic energy production [2,3]. However, prolonged exposure to hypoxia due to chronic lung diseases or residence at high altitudes leads to pulmonary vascular remodeling, which is a key pathological feature of pulmonary hypertension (group 3 PH due to lung diseases and/or hypoxia according to the classification of PH) [4,5,6]. PH is a progressive, incurable disease with multiple etiologies and is characterized by increased blood pressure within the arteries of the lungs [6]. One of the key events in pulmonary vascular remodeling is pulmonary arterial medial hypertrophy caused by increased proliferation and apoptotic resistance of pulmonary arterial smooth muscle cells (PASMCs) [7,8,9]. A growing body of evidence supports the concept of PH as a disease with cancer-like features [10,11,12]. Both cancer and PH pulmonary vascular resident cells, including PASMCs, undergo a metabolic shift towards glycolysis, either under hypoxia or even despite adequate oxygen (Warburg effect). Moreover, PH cells demonstrate abnormal activation of receptor tyrosine kinase (RTK) effector pathways, regulating cell growth, proliferation and survival, including the phosphatidylinositol 3-kinase/protein kinase B (PI3K/Akt) pathway. Interestingly, Akt was shown to mediate the induction of HIF-1α expression [13,14,15] and, together with adenosine monophosphate (AMP)-activated protein kinase (AMPK), to regulate the malignant phenotype of PASMCs [16]. The above findings suggest exploring the applicability of anti-cancer drugs to treat PH, including idiopathic pulmonary arterial hypertension (IPAH) [11]. The therapeutic concepts include direct or indirect inhibition of HIF activity and/or targeting growth and proliferation signaling pathways and their hubs [2,11,17].

Adenylate kinases catalyze the reversible interconversion of nucleotides, in particular adenine nucleotides (AMP, ADP, ATP), thereby functioning as a major hub in nucleotide-based metabolic and energetic signaling circuits [18,19]. In humans, nine adenylate kinase isoenzymes (AK1–AK9) are known, displaying different organ and subcellular distributions, kinetic properties, and functions in response to diverse physiological and pathological stimuli [19,20,21]. A rapidly growing body of research unveils the importance of AK isoenzymes in the regulation of numerous cellular processes, including cell differentiation and proliferation, motility, metabolic reprogramming, and cancer progression [22,23,24,25,26,27,28,29,30,31]. 

Adenylate kinase 4 (AK4) is localized to the mitochondrial matrix and functions as a stress-responsive protein, important for cell survival and proliferation [30,31,32,33,34]. The most extensive research on the function of this AK isoenzyme has been performed on cancer cells, where AK4 was shown to regulate hypoxia tolerance, cell proliferation and viability, mitochondrial activity, metastasis, and anti-cancer drug sensitivity [30,31,35]. Previous studies demonstrated that AK4 rewired the energy metabolism circuits to promote a glycolytic shift, leading ultimately to increased proliferation and tumor progression [30,35,36,37]. In lung cancer, the underlying mechanisms by which AK4 drives metastasis include the stabilization of HIF-1α and inhibition of AMPK [35]. Interestingly, the most recent study on murine M1 macrophages reported a novel role for AK4 in promoting inflammation, also via HIF-1α and AMPK signaling pathways [38]. HIF-1α was reported to transcriptionally regulate the expression of mouse AK4 [38,39,40]. The above findings uncover the existence of a vicious AK4-HIF-1α axis in cancer cells and macrophages and point to AK4 as a novel potential therapeutic target for malignancies and inflammatory disorders. 

Metabolic dysfunction is a common hallmark of cancer, inflammation and pulmonary hypertension [2,10,41,42,43,44,45]. AK4 is implicated in hypoxia responses and metabolic reprogramming in cancer; however, its function in normal (non-cancerous) human cells remains largely elusive. Thus, the pathophysiological role and the therapeutic potential of this protein in non-malignant proliferative disorders such as PH are still undiscovered. Against this background, our current study aimed to investigate the expression and function of AK4 in primary human PASMCs under normoxia and chronic hypoxia. We particularly focused on possible effects of AK4 on proliferation and energy metabolism of PASMCs in order to provide new insights into the altered cellular metabolism and cancer-like pathways in PH. 

## 2. Results

### 2.1. Hypoxia Upregulates AK4 in Primary Human Pulmonary Arterial Smooth Muscle Cells (PASMCs)

**Figure 1:**
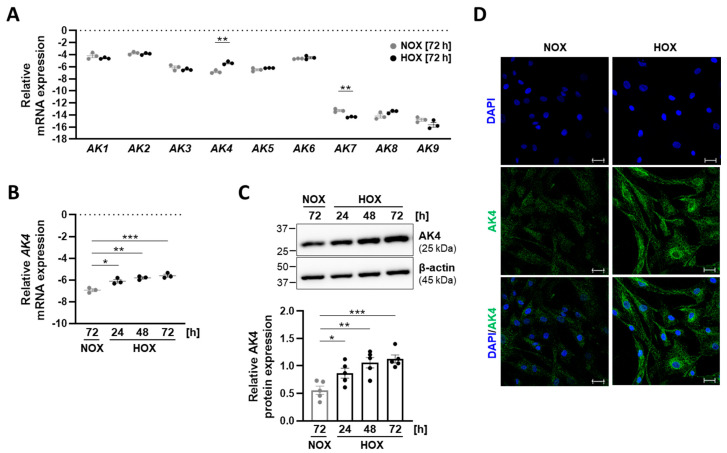
AK4 is upregulated in primary human pulmonary arterial smooth muscle cells (PASMCs) exposed to hypoxia. (**A**) Relative mRNA expression of *AK1*–*AK9* genes comprising the adenylate kinase family in PASMCs exposed to 21% O_2_ (normoxia, NOX) or 1% O_2_ (hypoxia, HOX) for 72 h; *n* = 3. (**B**) Relative mRNA expression of *AK4* in PASMCs exposed to normoxia (NOX) or hypoxia (HOX, 1% O_2_) for the indicated time points, compared to NOX; *n* = 3. (**C**) Representative Western blot for AK4 followed by densitometric quantification of relative AK4 expression in PASMCs exposed to normoxia (NOX) or hypoxia (HOX, 1% O_2_) for the indicated time points, compared to NOX; *n* = 5. (**D**) Immunocytochemical localization of AK4 (in green) in PASMCs cultured under normoxia (NOX) or hypoxia (HOX, 1% O_2_) for 48 h. Nuclear staining with DAPI (in blue). Scale bar: 50 µm. * *p* < 0.05, ** *p* < 0.01, *** *p* < 0.001, (**A**) unpaired Student’s *t*-test or (**B**,**C**) one-way ANOVA followed by Dunnett multiple comparisons test.

Until now, the gene expression patterns of the AK family members (*AK1*–*AK9*) and their potential regulation by hypoxia in human vascular smooth muscle cells (VSMCs) have not been investigated. In order to address this issue, we cultured primary human PASMCs under normoxia (21% O_2_) or chronic hypoxia (1% O_2_), followed by *AK1*–*AK9* mRNA expression analysis. PASMCs grown under normoxia exhibited markedly higher expression levels of *AK1*–*AK6* than *AK7*, *AK8*, and *AK9* (Figure 1A). Under hypoxia, the level of *AK7* was further reduced when compared to normoxia. Notably, among all the genes of the AK family, hypoxia exclusively upregulated *AK4*. To delineate a time-dependent regulation of *AK4* under hypoxia, we exposed PASMCs to 1% O_2_ for 24, 48, and 72 h. We found a time-dependent increase in AK4 both at the mRNA (Figure 1B) and protein (Figure 1C) levels. In addition, we consistently detected increased levels of AK4 in hypoxic PASMCs by immunocytochemistry (Figure 1D). 

### 2.2. AK4 Is Upregulated in Hypoxic PASMCs in a HIF-1α-Dependent Manner

**Figure 2:**
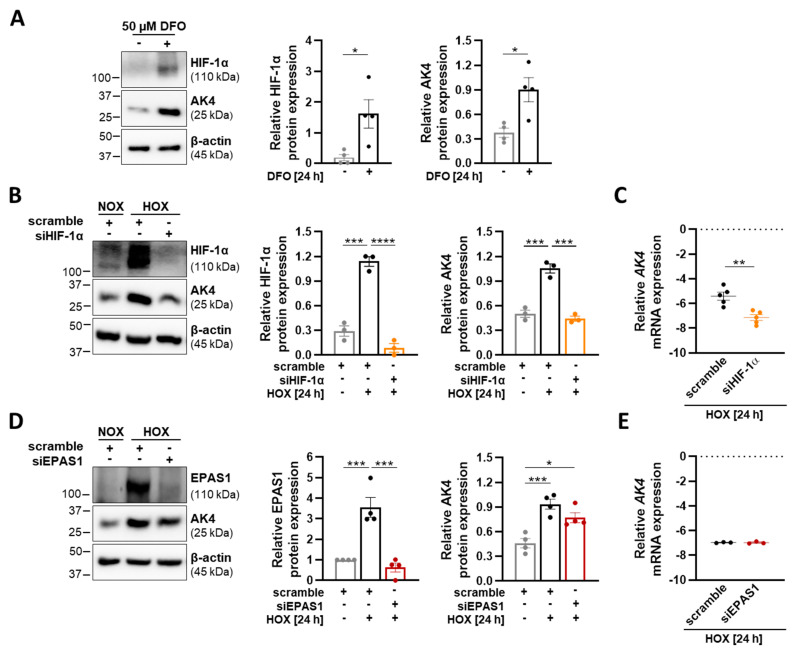
Hypoxia upregulates AK4 in an HIF-1α-dependent manner. (**A**) Representative Western blot for HIF-1α and AK4 followed by densitometric quantification of relative expression in PASMCs treated with 50 µM DFO for 24 h, compared to untreated control; *n* = 4. (**B**) Representative Western blot for HIF-1α and AK4 and densitometric quantification of relative expression in PASMCs transfected with HIF-1α siRNA (siHIF-1α), followed by hypoxic (HOX, 1% O_2_) exposure for 24 h, compared to non-targeting siRNA as a control (scrambled siRNA = scramble) under NOX or HOX; *n* = 3. (**C**,**E**) Relative mRNA expression of *AK4* in PASMCs transfected with siHIF-1α (**C**) or siEPAS1 (**E**), followed by hypoxic (HOX, 1% O_2_) exposure for 24 h, compared to control (scramble); *n* = 3–5. (**D**) Representative Western blot for EPAS1 (HIF-2α) and AK4 and densitometric quantification of relative expression in PASMCs transfected with EPAS1 siRNA (siEPAS1), followed by hypoxic (HOX, 1% O_2_) exposure for 24 h, compared to control (scramble) under NOX or HOX; *n* = 4. * *p* < 0.05, ** *p* < 0.01, *** *p* < 0.001, **** *p*< 0.0001, (**A**,**C**,**E**) unpaired Student’s *t*-test or (**B**,**D**) one-way ANOVA followed by Tukey multiple comparisons test.

Although HIF-1a is recognized to play a central role in the regulation of PASMC function, HIF-2α has recently been found to regulate PASMC stiffness contributing to pulmonary vascular remodeling [46]. Moreover, global partial HIF-2α gene deletion or pharmacological inhibition attenuates the initiation of hypoxia-induced PH [47]. Under hypoxia, prolyl hydroxylation of HIFs is inhibited, leading to its stabilization and nuclear translocation. To uncover the upstream signaling pathway(s) leading to the hypoxia-induced AK4 increase in PASMCs, we treated the cells with deferoxamine (DFO) under normoxic conditions. DFO is a well-known hypoxia-mimicking agent that stabilizes HIFs by inhibiting the activity of iron-dependent prolyl-hydroxylases (PHDs) through the depletion of iron [48]. We found a significant increase in HIF-1α and AK4 protein levels after DFO treatment, suggesting the involvement of HIFs in AK4 regulation (Figure 2A). In order to decipher the regulatory mechanism of the AK4 upregulation, we used the RNA interference approach to inhibit HIF-1α and EPAS1/HIF-2α signaling pathways. The siRNA-mediated knockdown of HIF-1α under hypoxia reduced the AK4 protein level to control (scramble) normoxic conditions (Figure 2B). siRNA against HIF-1α markedly reduced *AK4* mRNA expression in hypoxic PASMCs, indicating that AK4 is regulated by HIF-1α at the transcriptional level (Figure 2C). Knockdown of EPAS1/HIF-2α had no significant effect on the AK4 expression (Figure 2D,E). Collectively, the obtained results demonstrate that AK4 is a hypoxia-responsive protein in human PASMCs, and that HIF-1α acts as a dominant up-stream regulator of AK4 expression under hypoxia. 

### 2.3. AK4 Regulates the Viability and Proliferation of PASMCs under Both Normoxia and Hypoxia

**Figure 3:**
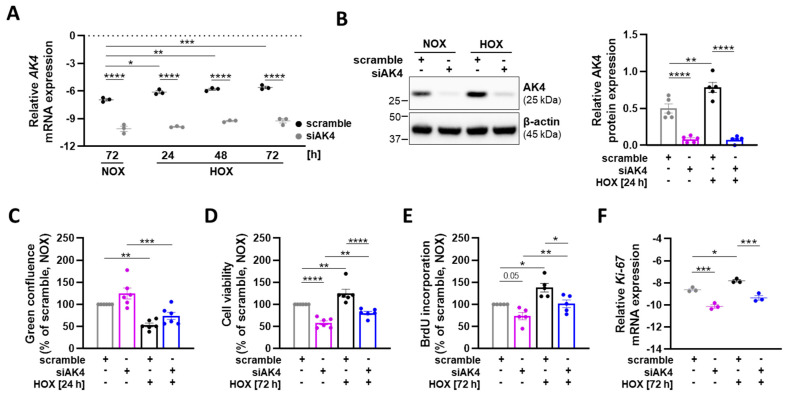
AK4 regulates the viability and proliferation of PASMCs both under normoxia and hypoxia. For all experiments, PASMCs were transfected with siRNA-targeting AK4 (siAK4) or non-targeting control (scrambled siRNA = scramble) followed by exposure to normoxia (NOX) or hypoxia (HOX, 1% O_2_) for the indicated time points. (**A**) Relative mRNA expression of *AK4* in PASMCs after AK4 knockdown (siAK4), compared to corresponding control (scramble) under NOX or HOX for 24, 48, and 72 h; *n* = 3. (**B**) Representative Western blot for AK4 followed by densitometric quantification of relative expression in PASMCs after AK4 knockdown (siAK4), compared to corresponding control (scramble) under NOX or HOX for 24 h; *n* = 5. (**C**) PASMC apoptosis measured by pSIVA-IANBD probe after AK4 knockdown (siAK4), compared to corresponding control (scramble) under NOX or 24 h HOX; *n* = 6. (**D**) PASMC viability measured by alamarBlue assay after AK4 knockdown (siAK4), compared to corresponding control (scramble) under NOX or HOX for 76 h; *n* = 6. (**E**) PASMC proliferation assessed by BrdU incorporation assay after AK4 knockdown (siAK4), compared to corresponding control (scramble) under NOX or HOX for 72 h; *n* = 5. (**F**) Relative mRNA expression of *Ki-67* in PASMCs after AK4 knockdown (siAK4) under normoxia (NOX) or hypoxia (HOX) for 72 h, compared to scramble; *n* = 3. * *p* < 0.05, ** *p* < 0.01, *** *p* < 0.001, **** *p* < 0.0001, two-way ANOVA followed by Bonferroni multiple comparisons test.

Chronic hypoxia triggers phenotypic alterations in PASMCs, including increased survival (apoptotic resistance) and proliferation, which cumulatively contribute to the process of pulmonary vascular remodeling [1,5]. To investigate the role of AK4 in the regulation of these pathophysiological functions of primary human PASMCs, the cells were transfected with siRNA-targeting AK4 (siAK4) or non-targeting siRNA as control (scramble), and subsequently cultured under either normoxia or hypoxia for the indicated time points. The applied RNA interference approach resulted in a significant knockdown of *AK4* mRNA both under normoxia and during the course of hypoxia (Figure 3A). Consistently, the silencing of AK4 led to a significant reduction in AK4 protein levels (Figure 3B). First, we studied whether AK4 is involved in regulation of PASMC apoptosis and survival. Although we did not find any significant changes in the apoptotic activity of PASMCs after AK4 knockdown compared to the corresponding controls (Figure 3C), we observed markedly lower viability of siAK4-transfected PASMCs under both normoxia and hypoxia (Figure 3D), which might be associated with altered cell proliferation. To address this issue, we analyzed the proliferation of PASMCs by quantifying the amount of BrdU (a thymidine analogue) incorporated into the newly synthesized DNA. BrdU labels the DNA of dividing cells only during the S-phase of the cell cycle. We found that PASMCs exposed to 72 h of hypoxia significantly increased the proliferation rate (BrdU incorporation), whereas the AK4 knockdown markedly reduced the cell proliferation under both normoxia and hypoxia (Figure 3E). Hypoxic PASMCs transfected with siAK4 exhibited a similar proliferative capacity to control (scramble) cells grown under physiological conditions (normoxia). In order to strengthen our findings, we analyzed the expression of Ki-67, which is a proliferation marker of all active cell cycle phases, except G0, thus providing more detailed information on cell proliferation response. We found reduced *Ki-67* mRNA expression in siAK4-transfected PASMCs under both normoxia and hypoxia (Figure 3F). To summarize, our data indicate that AK4 is involved in the regulation of viability and proliferation of human PASMCs. 

### 2.4. Downregulation of AK4 Augments the Mitochondrial Respiratory Function and Reduces the Glycolytic Metabolism in PASMCs

**Figure 4:**
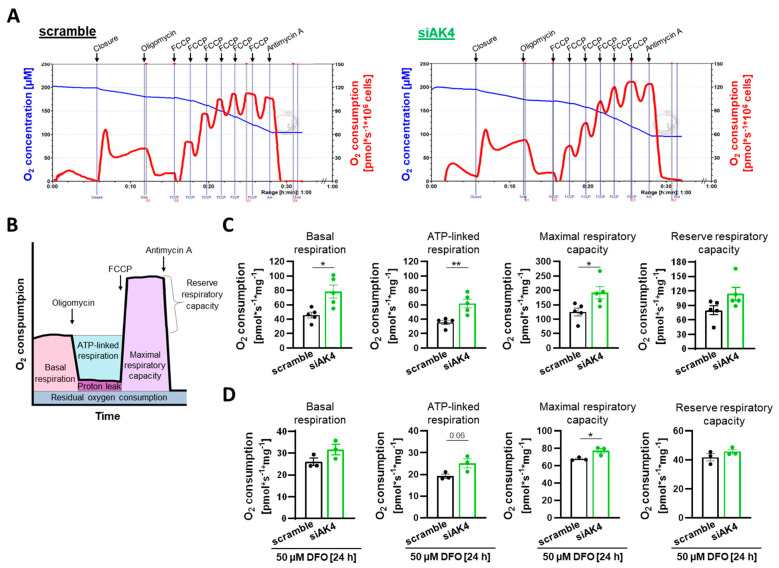
AK4 regulates mitochondrial respiratory function of PASMCs. (**A**) Representative graphs of high-resolution respirometry of intact PASMCs after transfection with control (left) scramble and siAK4 (right) treated with 4.5 µM oligomycin, sequential injections of FCCP (0.45 µM each), and 4.5 µM antimycin A. Measurements were performed with an Oroboros Oxygraph-2k instrument. The blue line indicates the oxygen concentration and the red line the oxygen flux per 10^6^ cells (O_2_ consumption). Gray vertical lines labeled 01, 02, 03, and 04 indicate the time points selected for quantification of respiration. (**B**) Schematic graph of O_2_ consumption rates after sequential addition of oligomycin, FCCP and antimycin A with depicted parameters of coupled and uncoupled respiration. (**C**) Mitochondrial respiration parameters of intact PASMCs after AK4 knockdown under normoxia, determined from the high-resolution respirometry measurements; *n* = 5. (**D**) Mitochondrial respiration parameters of intact PASMCs after AK4 knockdown followed by 50 µM DFO treatment for 24 h, determined from the high-resolution respirometry measurements; *n* = 3. * *p* < 0.05, ** *p* < 0.01, unpaired Student’s *t*-test.

A metabolic shift in energy production from mitochondrial oxidative phosphorylation (OXPHOS) to glycolysis contributes to excessive proliferation of PASMCs [10]. Owing to the fact that AK4 has been previously described to regulate the Warburg effect of human lung adenocarcinoma cells, we asked whether AK4 is also involved in the regulation of energy metabolism in non-malignant human vascular cells. First, using high-resolution respirometry, we studied the effect of AK4 knockdown on the mitochondrial respiration of primary human PASMCs. For this purpose, we measured oxygen consumption rates (OCR) after sequential injections of the following compounds: oligomycin, carbonyl cyanide *p*-(trifluoromethoxy) phenylhydrazone (FCCP), and antimycin A. Oligomycin inhibits ATP synthase and reduces OCR, FCCP is an uncoupler of mitochondrial oxidative phosphorylation which disrupts ATP synthesis and raises OCR to a maximal value, and antimycin A inhibits the electron transport chain and reduces OCR to a residual oxygen consumption, which refers to non-mitochondrial respiration [49]. We found that intact PASMCs transfected with siAK4 exhibited higher mitochondrial respiration under normoxia as compared to the control cells transfected with scramble siRNA (Figure 4A). The calculated OCR of coupled and uncoupled respiration (Figure 4B) showed that basal respiration, ATP-linked respiration, and maximal respiratory capacity were significantly increased after AK4 knockdown (Figure 4C). We found that treatment with 50 µM DFO for 24 h stabilizes HIF-1α protein in normoxic PASMCs (Figure 2A). Therefore, in the next step, we used this chemical approach to study PASMC respiratory function under hypoxia-mimicking conditions. The treatment with 50 µM DFO for 24 h prior the respirometry measurements resulted in a significant decrease in coupled and uncoupled respiration of PASMCs (*p* < 0.05) (Figure 4D). In these settings, we observed a tendency towards improving the PASMC mitochondrial respiration, in particular the maximal respiratory capacity, by AK4 knockdown (Figure 4D). 

**Figure 5:**
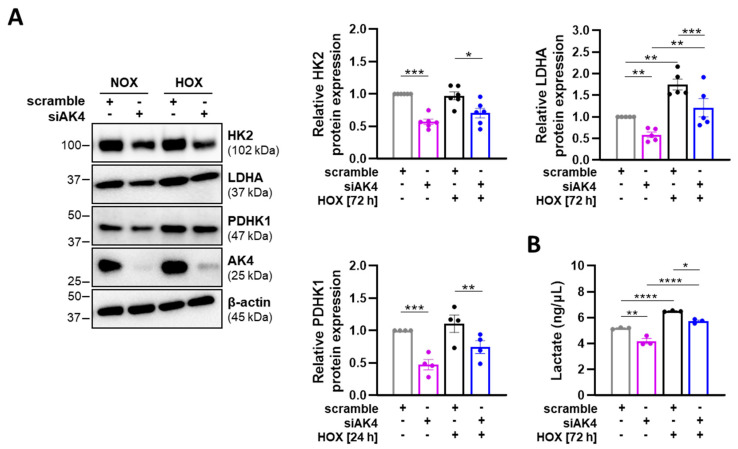
AK4 downregulation decreases the glycolytic metabolism in PASMCs. (**A**) Representative Western blots for HK2, LDHA, and PDHK1 followed by densitometric quantification of relative expression in PASMCs transfected with siAK4 followed by exposure to normoxia (NOX) or hypoxia (HOX, 1% O_2_) for 72 h (HK2 and LDHA detection) or 24 h (PDHK1 detection), compared to corresponding control (scrambled siRNA = scramble) under NOX or HOX; *n* = 4–6. (**B**) Lactate concentration in PASMC cultured media after AK4 knockdown followed by exposure to normoxia (NOX) or hypoxia (HOX, 1% O_2_) for 72 h, compared to corresponding control (scramble) under NOX or HOX; *n* = 3. * *p* < 0.05, ** *p* < 0.01, *** *p* < 0.001, **** *p* < 0.0001, two-way ANOVA followed by Bonferroni multiple comparisons test.

Finally, we found by Western blot that both normoxic and hypoxic PASMCs treated with siAK4 display significantly decreased expression levels of glycolytic enzymes, including hexokinase II (HK2) and lactate dehydrogenase A (LDHA), as well as pyruvate dehydrogenase kinase 1 (PDHK1), which regulates the pyruvate entry into the tricarboxylic acid (TCA) cycle (Figure 5A). Consistently, the level of extracellular lactate (the end product of anaerobic glycolysis) was significantly reduced in PASMCs after AK4 silencing, as compared to the corresponding control (scramble) cells (Figure 5B). These data indicate that AK4 is involved in energy metabolism of PASMCs, and its hypoxia-induced upregulation might be associated with a metabolic switch from OXPHOS to glycolysis.

### 2.5. AK4 Interacts with HIF-1α and Akt Signaling Pathways in PASMCs

**Figure 6:**
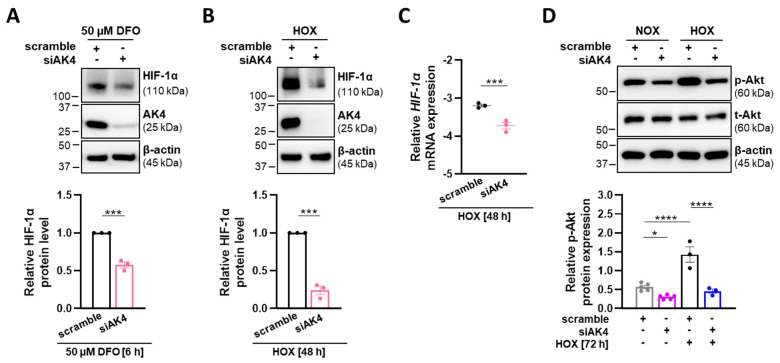
AK4 downregulation reduces HIF-1α levels and Akt signaling in PASMCs. (**A**,**B**) Representative Western blots for AK4 and HIF-1α and densitometric quantification of relative HIF-1α expression in PASMCs after AK4 downregulation followed by (**A**) treatment with 50 µM DFO for 6 h or (**B**) exposure to hypoxia (HOX, 1% O_2_) for 48 h; *n* = 3. (**C**) Relative mRNA expression of HIF-1α in PASMCs after AK4 knockdown (siAK4) under hypoxia (HOX, 1% O_2_) for 48 h, compared to control (scrambled siRNA = scramble); *n* = 3. (**D**) Representative Western blots for phospho-Akt (p-Akt) and total-Akt (t-Akt) followed by densitometric quantification of p-Akt levels in PASMCs transfected with siAK4 followed by exposure to normoxia (NOX) or hypoxia (HOX, 1% O_2_) for 72 h, compared to corresponding control (scramble) under NOX or HOX; *n* = 3–5. * *p* < 0.05, *** *p* < 0.001, **** *p* < 0.0001, (**A**–**C**) unpaired Student’s *t*-test or (**D**) two-way ANOVA followed by a Bonferroni multiple comparisons test.

HIF-1α and Akt play dominant roles in the development of hypoxia-induced PH [2,11]. Therefore, we investigated whether the observed reduction in proliferation, viability, and glycolytic shift after AK4 knockdown may be due to targeting HIF-1α and Akt signaling pathways. Indeed, AK4 silencing followed by both DFO treatment (Figure 6A) and hypoxia exposure (Figure 6B) resulted in significantly decreased HIF-1α protein levels. We also observed reduced *HIF-1α* mRNA expression in siAK4-transfected PASMCs (Figure 6C). Akt phosphorylation, which we found to be increased under hypoxia, was markedly reduced in PASMCs transfected with siAK4 when compared to the corresponding control (scramble) cells (Figure 6D). These observations indicate that AK4 is involved in regulation of HIF-1α and Akt signaling pathways. In addition, owing to the fact that hypoxia-induced AK4 upregulation is under the control of HIF-1α, our data suggest that AK4 and HIF-1α create a positive feedback loop in PASMCs.

### 2.6. AK4 Is Increased in the Lungs of IPAH Patients and Regulates the Glycolytic Metabolism of IPAH-PASMCs

**Figure 7:**
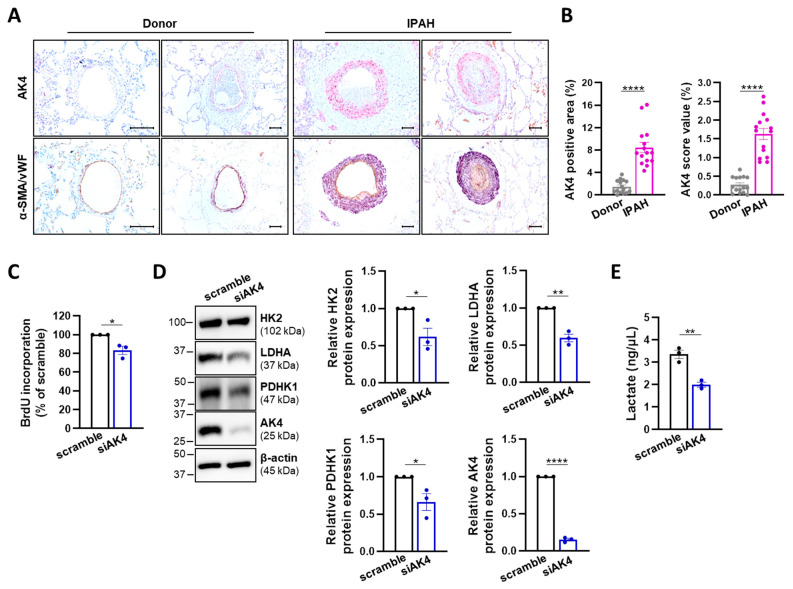
AK4 is increased in the pulmonary vasculature of IPAH patients and regulates glycolytic metabolism of IPAH-PASMCs. (**A**) Top: Representative images of immunohistochemical staining for AK4 (in pink) in donor and IPAH lungs; Bottom: Representative images of von Willebrand factor (vWF) (brown)-stained and α-smooth muscle actin (α-SMA) (violet)-stained lung tissue from donor and IPAH patients. Nuclear staining with hematoxylin (blue). Scale bar: 100 µm. (**B**) Quantification of AK4 immunohistochemical staining area and intensity in lung tissue from donor and IPAH patients; *n* = 15. (**C**) IPAH-PASMC proliferation assessed by BrdU incorporation assay after AK4 knockdown (siAK4), compared to control (scrambled siRNA = scramble); *n* = 3. (**D**) Representative Western blots for HK2, LDHA, PDHK1, and AK4 followed by densitometric quantification of their relative expression in IPAH-PASMCs transfected with siAK4 for 72 h, compared to control (scramble); *n* = 3. (**E**) Lactate concentration in IPAH-PASMC cultured media after AK4 knockdown for 72 h, compared to control (scramble); *n* = 3. * *p* < 0.05, ** *p* < 0.01, **** *p* < 0.0001, unpaired Student’s *t*-test.

A pro-proliferative and glycolytic phenotype of PASMCs is a hallmark of both hypoxia-induced PH and idiopathic pulmonary arterial hypertension (IPAH) [41]. We performed AK4 immunohistochemical staining in the lung tissue of healthy subjects (donors) and from patients with IPAH and observed a significant increase in both area and intensity of the AK4 signal in lungs from IPAH patients (Figure 7A,B). AK4 was predominantly localized in alpha-smooth muscle actin (α-SMA)-positive cells within the vessel wall (Figure 7A). In view of our above findings on the role of AK4 in donor PASMCs, we investigated whether AK4 silencing leads to similar effects in PASMCs derived from IPAH patients. Indeed, AK4 knockdown decreased the cell proliferation (Figure 7C), protein expression of HK2, PDHK1, and LDHA (Figure 7D), and accordingly reduced the accumulation of extracellular lactate (Figure 7E) in IPAH-PASMCs, as compared to control (scramble) cells. These observations suggest that AK4 may be involved in the regulation of cell proliferation and glycolytic metabolism of PASMCs from distinct PH entities.

## 3. Discussion

Our study provides, to the best of our knowledge, the first evidence for (1) a hypoxia-dependent upregulation of AK4 in primary human non-malignant cells, namely pulmonary arterial smooth muscle cells, and (2) the role of this enzyme in the regulation of PASMC proliferation and energy metabolism under both physiological and pathophysiological (chronic hypoxia) conditions. Moreover, we (3) found elevated AK4 levels in α-SMA-positive cells in remodeled pulmonary vessels from IPAH patients, and reduced proliferation and glycolytic metabolism of IPAH-PASMCs after AK4 knockdown, supporting a potential in vivo role of AK4 in the process of pulmonary vascular remodeling. 

Previous studies demonstrated differential regulation of AK4 expression under various stress conditions. Hypoxia was reported to upregulate AK4 in human cancer cell lines such as HeLa (cervical cancer), A549 (lung carcinoma), and SHSY5Y (neuroblastoma) [30,33,35] as well as in mouse chondroprogenitor cells and immortalized HEK293 (human embryonic kidney) cells [32,33,50]. Interestingly, AK4 was downregulated by hypoxia in a human liver cancer cell line, HepG2. This downregulation was explained by markedly higher basal levels of AK4 in HepG2 cells as compared to HEK293, and the disparate hypoxia responses of these two cell lines [32]. Other studies reported increased AK4 expression in SHSY5Y and VSMCs under oxidative stress [33,51] and in rat liver after administration of hepatotoxicans such as acetaminophen, amiodarone, tetracycline, and carbon tetrachloride [52]. In our work, we demonstrated that chronic hypoxia increased mRNA and protein levels of AK4, predominantly via HIF-1α-dependent signaling. These findings are in agreement with previous studies reporting the reduced expression of AK4 mRNA or protein after genetic ablation of HIF-1α in murine macrophages [38], stem cells [39], and fibroblasts [40], or increased AK4 protein levels upon the administration of HIF-1α stabilizers such as deferoxamine (DFO) or dimethyloxalylglycine (DMOG) in HeLa cells [30] and murine macrophages [38], respectively. We found no significant changes in AK4 mRNA and protein expression after EPAS1 silencing, suggesting that HIF-2α is not involved in hypoxia-driven AK4 upregulation in human PASMCs.

Although PH is a non-malignant disease, increasing evidence indicates that it shares numerous similarities with cancer. Thus, the knowledge gained from cancer research may help in understanding the pathobiology of PH and in developing new treatment strategies thereof [10,11,12]. The augmented proliferation and glycolysis are common hallmarks shared between cancer cells and PH-PASMCs. In cancer, AK4 upregulation is associated with metabolic reprogramming, tumor progression, and metastasis [30,35,36]. In our study, siRNA-mediated AK4 knockdown decreased PASMC viability and proliferation, improved mitochondrial respiratory function, and reduced glycolytic metabolism (decreased levels of HK2, LDHA, PDHK1, and lactate). These findings are again in line with observations in cancer cells, where AK4 knockdown improved mitochondrial activity (increased maximal respiratory capacity after FCCP treatment) and prevented a glycolytic shift (decreased HK2 and lactate levels), leading ultimately to reduced proliferative/metastatic potential [30,35]. In our study, we observed a similar impact of AK4 silencing on cell survival, proliferation, and energy metabolism both under hypoxic and normoxic conditions. This indicates that AK4 plays an essential role in PASMCs not only by regulating cellular responses to hypoxia but also under physiological conditions. Similar effects of AK4 targeting have been described for immortalized human embryonic kidney cells [33] and some cancer cell lines [28,29,37]. This points to a common fundamental role of AK4 in cancer cells and PASMCs. 

In our study, we provided evidence that AK4 may regulate the activation status of Akt and form a positive feedback loop with HIF-1α, thereby contributing to the development of a pro-proliferative, pro-survival, and glycolytic phenotype of PASMCs. The existence of an AK4-HIF-1α feedforward loop has been recently shown in a human lung adenocarcinoma cell lines [35] and in murine M1 macrophages [38]. In lung cancer, AK4 operates as an upstream HIF-1α regulator by stabilizing HIF-1α through the inhibition of its hydroxylation and subsequent degradation [35], whereas in macrophages it acts by enhancing both HIF-1α gene transcription and protein stability [38]. Our study suggests that AK4 regulates HIF-1α levels in a similar fashion, as observed in macrophages. In this regard, we found that AK4 silencing under hypoxia results in HIF-1α downregulation at both the mRNA and protein levels as well as in the reduction in the HIF-1α protein amount upon treatment with DFO—an agent that induces the accumulation of HIF-1α by inhibition of its hydroxylation. To the best of our knowledge, there are no reports about a mutual interaction between AK4 and HIF-1α proteins, but previous studies in human cancer cell lines [35] and murine macrophages [38] showed that AK4 contributes to HIF-1α stabilization via increasing the production of cytosol and/or mitochondrial reactive oxygen species (ROS). Since we found that AK4 has a similar function both in non-malignant cells (PASMCs) and lung cancer cells, we can speculate that this might be one possible mechanism underlying the AK4-mediated HIF-1α increase observed in human PASMCs. Notably, we found reduced levels of HIF-1α and phospho-Akt in siAK4-treated PASMCs, suggesting that AK4 may regulate HIF-1α stabilization via Akt signaling. This concept is supported by previous studies showing that Akt acts as an upstream mediator of HIF-1α activation in cancer cells [53] and PASMCs [13,14,15,16], leading ultimately to increased cell survival, proliferation, and a glycolytic switch. Targeting the AK4-HIF-1α axis has been proven to be a potent therapeutic strategy in non-small-cell lung carcinoma [35]. In view of the recently proposed therapeutic approaches for the treatment of PH (targeting HIF-1α, RTK signaling pathways and/or their hubs) [2,11,17], our study raises the possibility that AK4 may be a new potential target in PH treatment. 

**Figure 8:**
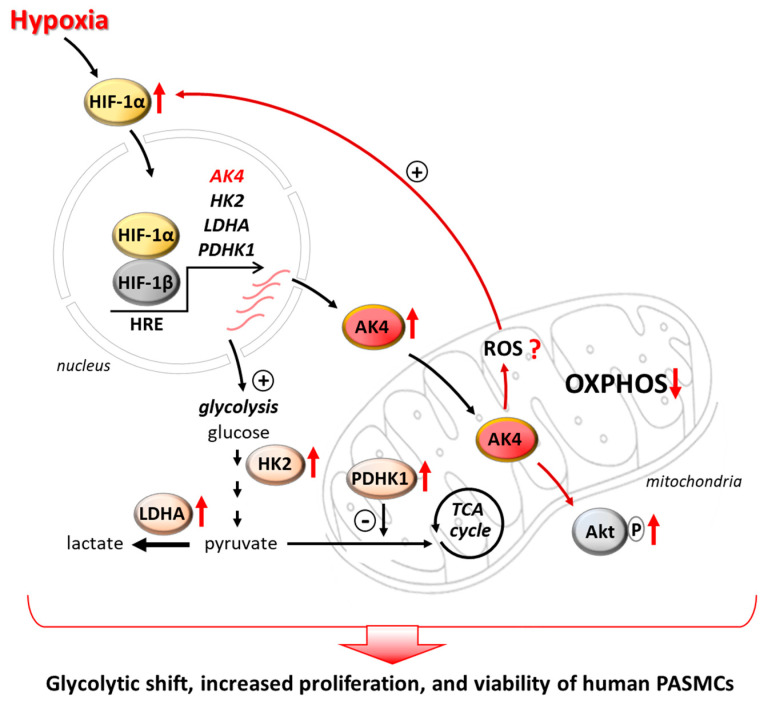
Schematic diagram depicting a possible role of AK4 in promoting a glycolytic, pro-proliferative, and pro-survival phenotype of PASMCs. Hypoxia upregulates AK4 in an HIF-1α-dependent manner. AK4 upregulation leads to Akt activation and increases HIF-1α levels, possibly by increasing ROS, indicating the existence of an AK4-HIF-1α feedforward loop in hypoxic PASMCs. The AK4-HIF-1α axis gives rise to increased levels of glycolytic enzymes (HK2, LDHA, PDHK1), promotes a metabolic shift from OXPHOS towards glycolysis (glycolytic shift), and increases PASMC proliferation and viability. This abnormal phenotype of PASMCs is a hallmark of the pulmonary vascular remodeling in pulmonary hypertension. Thus, targeting AK4 may represent a new therapeutic strategy for PH.

In conclusion, our findings indicate that AK4 acts as a new hypoxia-responsive metabolic regulator in PASMCs, controlling cell proliferation, viability, and a glycolytic shift (Figure 8). Modulation of AK4 expression or activity could provide a novel therapeutic tool for targeting the pulmonary vascular remodeling, which is a hallmark of PH. However, further studies are needed to (1) unveil the mechanisms underlying the AK4-dependent regulation of HIF-1α and Akt, (2) investigate the in vivo role of AK4 in the pathogenesis of PH, and (3) develop pharmacological strategies targeting AK4 to assess its therapeutic potential for different clinical groups of PH. 

## 4. Materials and Methods

### 4.1. Human Lung Samples

Lung tissue was obtained from explanted lungs from patients with or without idiopathic pulmonary arterial hypertension (IPAH) obtained from the UGMLC Giessen Biobank of the Justus-Liebig University Giessen (Germany). The explanted lung tissue was fixed in 4% (*w*/*v*) paraformaldehyde (PFA) in phosphate-buffered saline (PBS) and stored at 4 °C until sectioning and immunohistochemical staining. The studies were approved by the Ethics Committee of the Justus-Liebig-University School of Medicine (AZ 10/06, 58/15).

### 4.2. Cell Culture and Hypoxia Exposure of PASMCs

Primary human PASMCs isolated from healthy individuals without pulmonary hypertension (donors) were purchased from Lonza (Basel, Switzerland). PASMCs from IPAH patients were obtained from the UGMLC Giessen Biobank of the Justus-Liebig University Giessen (Germany). The collection of the human material was performed in accordance with the protocol approved by the Ethics Committee of the Justus-Liebig-University School of Medicine (AZ 10/06, 58/15).

Human PASMCs were grown in Smooth Muscle Growth Medium 2 (Cat# C-22062, Promocell, Heidelberg, Germany) with 50 U/mL penicillin and 50 µg/mL streptomycin as antibiotics (Thermo Fisher Scientific, Dreieich, Germany). Cells were maintained at 37 °C in a humidified normoxic atmosphere containing 21% O_2_ and 5% CO_2_. Hypoxia exposure was performed under normobaric conditions at 37 °C in a cell culture incubator (Heracell, Thermo Fisher Scientific, Dreieich, Germany) equilibrated with a water-saturated gas mixture of 1% O_2_, 5% CO_2_, and 94% N_2_, equipped with an oxygen sensor to monitor oxygen levels. Human PASMCs were incubated in the growth medium under normoxia (NOX) or hypoxia (HOX) for the indicated time points. Alternatively, hypoxia was mimicked by PASMC treatment with 50 µM of DFO (deferoxamine mesylate salt; #Cat D9533, Sigma Aldrich, Munich, Germany), a well-known HIF-1α stabilizer [48]. Cells at passages 3 to 7 were used in the experiments. In the case of hypoxia experiments, all sample preparations and assays were conducted and terminated directly in the hypoxic chamber through the tightly adjusted sleeves by using appropriate buffers and reagents pre-equilibrated at 37 °C in 1% O_2_ atmosphere for at least 6 h. 

### 4.3. RNA Interference by Synthetic siRNA

Human PASMCs were transfected with synthetic siRNA-targeting AK4 (siAK4, Cat# L-006700-00), HIF-1α (siHIF-1α, Cat# L-004018-00), or EPAS1/HIF-2α (siEPAS1, Cat# L-004814-00) purchased from Dharmacon (Lafayette, CO, USA), or with non-targeting siRNA as a control (scramble, Cat# SR-CL000-005), purchased from Eurogentec (Luettich, Belgium). Cells were transfected in the growth medium with 100 nM siRNA using 0.5 µL of Lipofectamine 3000 Transfection Reagent (Thermo Fisher Scientific, Dreieich, Germany) per 1 cm^2^ surface area of the culture vessel, according to the manufacturer’s protocol. Six hours after siRNA transfection, the new growth medium was changed, and cells were cultured for the next 72 h under normoxia or hypoxia for the indicated time points. 

### 4.4. Cell Viability Assay

Human PASMCs were seeded on 96-well plates at a density of 8000/cm^2^. The next day, cells were transfected with scramble or siAK4 as described above and exposed to normoxia or hypoxia for 72 h. The viability of PASMCs was assessed using alamarBlue Cell Viability Reagent (Cat# DAL1025, Thermo Fisher Scientific, Dreieich, Germany), according to the manufacturer’s instructions. Briefly, the alamarBlue reagent was diluted 1:10 with the growth medium that was pre-incubated for 6 h at 37 °C in a cell culture incubator under normoxic or hypoxic conditions. After the addition of the alamarBlue reagent, cells were incubated for up to 4 h at 37 °C under normoxia or hypoxia. The fluorescence was measured in a Tecan Infinite 200 PRO multimode plate reader (Männedorf, Switzerland), using excitation at 560 and emission at 600 nm. The cell viability was plotted as a percentage of absorbance compared to the normoxia control (scramble, NOX).

### 4.5. Apoptosis Assay

Human PASMCs were seeded on 96-well plates at a density of 8000/cm^2^. The next day, cells were transfected with scramble or siAK4 as described above and grown under normoxia for 48 h. For the assessment of PASMC apoptosis, an Annexin XII-based polarity-sensitive probe pSIVA-IANBD (from Kinetic Apoptosis Kit, Cat# ab129817, Abcam, Berlin, Germany) was used according to the manufacturer’s instructions. After addition to the cells, pSIVA-IANBD fluorescence was measured after 24 h under normoxic or hypoxic conditions, using the Incucyte ZOOM Live-Cell Analysis System (Essen Bioscience, Ann Arbor, MI, USA). The results were calculated by normalizing the green object confluence metric to phase object confluence metric and plotted as the percentage of green confluence compared to the normoxic control (scramble, NOX).

### 4.6. Cell Proliferation Assay

Human PASMCs were seeded on 24-well plates at a density of 6000/cm^2^. The next day, cells were serum-starved for 24 h in Smooth Muscle Basal Medium 2 (Cat# C-22262, PromoCell, Heidelberg, Germany) with antibiotics, followed by siRNA transfection as described above. Afterwards, cells were exposed to normoxia or hypoxia for 72 h. The effect of AK4 knockdown on PASMC proliferation was assessed using a Cell Proliferation Elisa BrdU (bromodeoxyuridine) Colorimetric Kit (Cat# 11647229001, Roche Diagnostics, Mannheim, Germany), according to the manufacturer’s protocol. Cells were incubated with a BrdU labeling solution for 18 h. The absorbance was measured at 370 nm with reference at 492 nm in a Tecan Infinite 200 PRO multimode plate reader. The cell proliferation was plotted as the percentage of absorbance compared to the normoxia control (scramble, NOX). 

### 4.7. Lactate Production Assay

Human PASMCs were seeded on 6-well plates at a density of 6000/cm^2^. The next day, cells were transfected as described above and then exposed to hypoxia for 72 h. Control cells were maintained under normoxic conditions. Afterwards, the culture media were collected, centrifuged for 5 min at 300× *g* at 4 °C, and the obtained supernatants were snap-frozen in liquid nitrogen and stored at −80 °C till the measurement of lactate concentrations could be performed. Lactate concentration was measured using the Lactate Assay Kit (Cat# MAK064, Sigma Aldrich, Munich, Germany) according to the manufacturer’s instructions. The absorbance was measured at 570 nm in a Tecan Infinite 200 PRO multimode plate reader and the lactate concentration was determined from the lactate calibration curve.

### 4.8. Measurement of PASMC Mitochondrial Respiration Using High-Resolution Respirometry

Human PASMCs were seeded on 10 cm culture dishes at a density of 6000/cm^2^ and grown until they reached 70% confluency. Then, cells were transfected with scramble or siAK4 as described above and cultured under normoxia for 72 h with or without treatment with 50 µM DFO within the final 24 h. The effect of AK4 knockdown on PASMC mitochondrial respiratory function was assessed by measuring the oxygen consumption rate of intact cells using an Oxygraph-2k instrument (Oroboros Instruments, Innsbruck, Austria). Seventy-two hours after siRNA transfection, PASMCs were washed once with PBS, trypsinized, and centrifuged at 220× *g* for 5 min. The obtained cell pellet was resuspended in 2 mL of pre-warmed PASMC growth medium containing 20 mM HEPES-NaOH pH 7.0 (without Ca^2+^ and Mg^2+^ ions). Cells were counted using a Neubauer hemocytometer and diluted in the above-described respiration buffer to a final volume of 2.2 mL and a density of 0.6 − 1 × 10^6^ cells/mL. The Oxygraph-2k instrument was calibrated by performing a background calibration and air calibration according to the manufacturer’s recommendations (Oroboros Instruments, Innsbruck, Austria). PASMC mitochondrial respiration was measured at 37 °C. Cells were transferred into the two stirred chambers of the Oxygraph-2k instrument, which, after equilibration, were sealed to obtain a closed system. Decreasing oxygen concentration in the chambers resembled cellular oxygen consumption. First, steady-state oxygen consumption displaying “routine” respiration was measured. Subsequently, cells were injected with 5 mM oligomycin (Cat# O4876, Sigma Aldrich, Munich, Germany) to a final concentration of 4.5 µM to inhibit the ATP synthesis, which is coupled to the activity of the electron transport chain (detection of proton leak). In a following step, the maximal respiratory capacity of the mitochondrial electron transport system (ETS) was measured by sequential injections (titration) of 5 mM uncoupler carbonyl cyanide p-(trifluoromethoxy) phenylhydrazone (FCCP, Cat# C2920, Sigma-Aldrich, Munich, Germany) to a final concentration of 0.45 µM each. After reaching the maximal respiration, 5 mM antimycin A (Cat# A8674, Sigma-Aldrich, Munich, Germany) was injected into the chambers to a final concentration of 4.5 µM to inhibit mitochondrial respiration (residual oxygen consumption). The applied protocol allowed us to determine the following parameters of coupled and uncoupled respiration of the cells: basal respiration, ATP-linked respiration, maximal respiratory capacity, reserve (spare) respiratory capacity, and proton leak, corrected to residual oxygen consumption [49]. The analysis of the oxygen consumption of the respiratory states was performed using DatLab 5.1 software (Oroboros Instruments, Innsbruck, Austria). The oxygen consumption rates expressed as picomoles per second per 1 × 10^6^ cells were normalized to the total cellular protein content. The total protein in a sample was determined using a bicinchoninic acid (BCA) assay kit (Pierce, Rockford, IL, USA) according to the manufacturer’s protocol. 

### 4.9. Immunocytochemistry

Human PASMCs were seeded on chamber slides at a density of 6000/cm^2^ and the next day, exposed to normoxia or hypoxia for 48 h. Cells were fixed with 4% PFA for 10 min at room temperature (RT), washed 3 times for 5 min with PBS, and blocked for 1 h with 3% serum bovine albumin (BSA) in PBS supplemented with 1% Triton X-100. Next, cells were incubated overnight at 4 °C with rabbit anti-AK4 primary antibody (Cat# 30038, GeneTex, Irvine, CA, USA), diluted 1:150 in PBS containing 3% BSA and 0.1% Triton X-100. Afterwards, cells were washed 3 times with PBS for 5 min and incubated for 1.5 h at RT with Alexa Fluor Plus 488 secondary antibody (Cat# A32790, Thermo Fisher Scientific, Dreieich, Germany) diluted 1:400 in PBS containing 3% BSA and 0.1% Triton X-100. Cell nuclei were counterstained with 4′,6-diamidine-2′-phenylindole dihydrochloride (DAPI). Finally, slides were mounted with a fluorescent mounting medium (Dako). Fluorescent imaging was performed with a confocal microscope (Leica SP5, Leica Microsystems, Wetzlar, Germany).

### 4.10. Immunohistochemistry

For AK4 localization and quantification in human lungs, paraffin-embedded tissue sections were deparaffinized and rehydrated as previously described [54]. Endogenous peroxidases were blocked with 3% hydrogen peroxide solution (Sigma-Aldrich, Munich, Germany) in methanol for 20 min. Antigen retrieval was performed by cooking the slides in HIER Citrate Buffer pH 6.0 (Cat# ZUC028, Zytomed Systems, Berlin, Germany). After blocking with 10% BSA for 1 h at RT, tissue sections were incubated with Blocking Solution (ZytoChem Plus AP Polymer Kit, Cat# POLAP, Zytomed Systems, Berlin, Germany) according to the manufacturer’s instructions, followed by overnight incubation at 4 °C with rabbit anti-AK4 primary antibody at a dilution of 1:100 (Cat# HPA042753, Prestige Antibodies^®®^ Powered by Atlas Antibodies, Sigma Aldrich, Munich, Germany). After that step, tissue sections were incubated with the enhancement reagent PostBlock followed by AP-Polymer (from ZytoChem Plus AP Polymer Kit, Zytomed Systems, Berlin, Germany), according to the manufacturer’s instructions. The bound antibody was detected using Warp Red Chromogen Kit (Cat# WR806, Biocare Medical, Pacheco, CA, USA), according to the manufacturer’s protocol. The tissue was counterstained with hematoxylin. The AK4 staining intensity in the lung tissue was quantified with a light microscope using the Qwin software (Leica Microsystems, Wetzlar, Germany).

### 4.11. RNA Isolation and RT-qPCR 

Total RNA was isolated from PASMCs using the RNeasy Mini Kit (Qiagen, Hilden, Germany) according to the manufacturer’s instructions. The purity and concentration of isolated RNA was measured using a NanoDrop ND-1000 spectrophotometer (Thermo Fisher Scientific, Dreieich, Germany). The RNA purity with the A_260_/A_280_ ratio of 1.8–2.0, A_260_/A_230_ of 2.0–2.2 was considered for the qPCR analysis. An amount of 1 µg of RNA was used for the first-strand cDNA synthesis using the iScript cDNA Synthesis Kit (Bio-Rad, Munich, Germany). For the quantitative real-time polymerase chain reaction (qPCR), an iQ SYBR Green Supermix (BioRad, Munich, Germany) was used according to the manufacturer’s protocol. The qPCR analysis was carried out in a CFX Connect™ Real-Time PCR Detection System (Bio-Rad, Munich, Germany) as previously described [55]. A total of 10 ng of cDNA was used as a template for qPCR. Human gene-specific primers were designed based on the sequence information from the NCBI database and purchased from Metabion (Marinsried, Germany). The relative gene expression levels were calculated using the ΔCt method. The housekeeping gene encoding beta-2-microglobulin (B2M) was used as the internal reaction control to normalize the Ct values of the target genes using the following equation: ΔCt = Ct_reference gene_ − Ct_target gene_. The primer sequences used in this study are listed in Table S1. 

### 4.12. Western Blot

The protein isolation from PASMCs and Western blot analysis was carried out as previously described [55]. The polyvinylidene fluoride (PVDF) membranes were incubated overnight at 4 °C with the following primary antibodies: rabbit anti-AK4 (diluted 1:1000; Cat# 30038, GeneTex, Irvine, CA, USA), mouse anti-HIF-1α (1:300; Cat# 6109, BD Biosciences, San Jose, CA, USA), goat anti-EPAS1 (1:500; Cat# AF2997, R&D Systems, Wiesbaden-Nordenstadt, Germany), rabbit anti-phospho-Akt (1:1000; Cat# 9271, Cell Signaling Technology, Danvers, MA, USA), rabbit anti-Akt (1:1000; Cat# 9272, Cell Signalling Technology, Danvers, MA, USA), rabbit anti-HK2 (1:1000; Cat# ab209847, Abcam, Berlin, Germany), rabbit anti-LDHA (1:1000; Cat# 2012, Cell Signaling Technology, Danvers, MA, USA), rabbit anti-PDHK1 (1:1000; Cat# 3820, Cell Signaling Technology, Danvers, MA, USA), and mouse anti-β-actin (1:50,000; Cat# A2228, Sigma-Aldrich, Munich, Germany). The horseradish-peroxidase (HRP)-labeled secondary antibodies anti-rabbit (Cat# W4021) and anti-mouse (Cat# W4011) were purchased from Promega (Madison, WI, USA) and used at a dilution of 1:5000. The HRP-conjugated anti-goat antibody (Cat# HAF109) was purchased from R&D Systems Wiesbaden-Nordenstadt, Germany) and used at 1:1000 dilution. Bound antibodies were detected by chemiluminescence in the ChemiDoc Touch Imaging System (BioRad, Munich, Germany) using Clarity™ Western ECL Blotting Substrate (BioRad, Munich, Germany). The chemiluminescent Western blots were quantified by Image Lab Software (BioRad, Munich, Germany). Band intensity values were normalized to β-actin, which was used as a loading control. 

### 4.13. Statistical Analysis

The data are expressed as mean ± standard error of mean (SEM). The results were obtained from at least three biological replicates. To determine the statistical significance, one-way analysis of variance (ANOVA) followed by a Dunnett or Tukey post hoc test for multiple comparisons and an unpaired Student’s *t*-test for two group comparisons were used. Grouped comparisons were performed using two-way ANOVA followed by Bonferroni’s multiple comparisons test. Statistical analysis and data visualization were performed with GraphPad Prism 9.2 software (GraphPad Software, San Diego, CA, USA). A *p*-value of less than 0.05 was considered statistically significant.

## Data Availability

All data were reported in the manuscript and in the Supplementary Materials.

## Supplementary Materials

The following are available online at https://www.mdpi.com/article/10.3390/ijms221910371/s1.

## Abbreviations

AK4	adenylate kinase 4
Akt	protein kinase B
B2M	beta-2-microglobulin
BrdU	bromodeoxyuridine
DFO	deferoxamine
EPAS1	endothelial PAS domain-containing protein 1 (HIF-2α)
FCCP	carbonyl cyanide 4-(trifluoromethoxy)phenylhydrazone
HIF	hypoxia inducible factor
HK2	hexokinase 2
HOX	hypoxia
IPAH	idiopathic pulmonary arterial hypertension
LDHA	lactate dehydrogenase A
Ki-67	marker of proliferation Ki-67
NOX	normoxia
OCR	oxygen consumption rate
OXPHOS	oxidative phosphorylation
PASMCs	pulmonary arterial smooth muscle cells
PBS	phosphate-buffered saline
PDHK1	pyruvate dehydrogenase kinase 1
PH	pulmonary hypertension
RT	room temperature
RT-qPCR	reverse transcription quantitative polymerase chain reaction
scramble	scrambled siRNA (control siRNA)
TCA	tricarboxylic acid
VSMCs	vascular smooth muscle cells
