# Supplementary material for: Adenylate Kinase 4—A Key Regulator of Proliferation and Metabolic Shift in Human Pulmonary Arterial Smooth Muscle Cells via Akt and HIF-1α Signaling Pathways
Source: Int J Mol Sci. 2021 Sep 26;22(19):10371. doi: 10.3390/ijms221910371 (PMC8508902; doi:10.3390/ijms221910371)
Supplement: Supplementary file 1 [file ijms-22-10371-s001.zip › ijms-1392179-supplementary.pdf]

Table S1. Detailed information on primers used in this study.

Gene Name	GenBank Accession Number	Forward primer	Reverse primer
AK1	NM_000476.2	5’-GGCTTCCTGATTGATGGCT-3’	5’-ATGGTCTCCTCATTGTCGTCC-3’
AK2	NM_001625.3	5’-ACTGTGAGGCAGGCAGAAATG-3’	5’-ATGGGCTCTTTTGGAGGGTTG-3’
AK3	NM_016282.3	5’-TCGCGCATCACTACACACTT-3’	5’-TCTGGGATGAGTTTCCCTTGGT-3’
AK4	NM_001005353.3	5’-TCTTGCGGGAGAACATCAAGG-3’	5’- CGGACATCATTAGGCGTGTG-3’
AK5	NM_174858.2	5’-GAGGTGAAGCAAGGGGAAGAG-3’	5’-CTTTGGAGAAGGCGGTTGGT-3’
AK6	NM_016283.4	5’-GATTTCTTCCCTGAACGCTGG-3’	5’-CCTTGTAGGATGCTGTGGCTT-3’
AK7	NM_152327.4	5’-GGCGAGATTCCTGCATTACCA-3’	5’-CAGACTCATCCACAGCAACCA-3’
AK8	NM_152572.2	5’-GGTCATTCCCTCCTACCCCAA-3’	5’-AGACTTTTCCCACTGCCCAC-3’
AK9	NM_001145128.2	5’-ATATCCGCCAACCCAAACCT-3’	5’-GCTCTGTTTCCGGGTGATTGT-3’
B2M	NM_004048.4	5’-GCCGTGTGAACCATGTGACT-3’	5’-GCAAGCAAGCAGAATTTGGA-3’
HIF-1α	NM_001530.4	5’-TTACAGCAGCCAGACGATCATG-3’	5’-TGGTCAGCTGTGGTAATCCACT-3’
EPAS1	NM_001430.5	5’-CTGATGGCCATGAACAGCATCT-3’	5’-TCCTCGAAGTTCTGATTCCCGA-3’
Ki67	NM_002417.5	5’-GCAAGCACTTTGGAGAGC-3’	5’-TCTTGACACACACATTGT-3’
